# Arginine Supplementation in MELAS Syndrome: What Do We Know about the Mechanisms?

**DOI:** 10.3390/ijms25073629

**Published:** 2024-03-24

**Authors:** Camila D. S. Barros, Aryane Coutinho, Celia H. Tengan

**Affiliations:** Division of Neurology, Department of Neurology and Neurosurgery, Escola Paulista de Medicina, Universidade Federal de São Paulo, São Paulo 04039-032, Brazil; dantas.ca@hotmail.com (C.D.S.B.); aryanescoutinho@gmail.com (A.C.)

**Keywords:** MELAS, mitochondrial DNA, mitochondria, arginine, oxidative stress, nitric oxide

## Abstract

MELAS syndrome, characterized by mitochondrial myopathy, encephalopathy, lactic acidosis and stroke-like episodes, represents a devastating mitochondrial disease, with the stroke-like episodes being its primary manifestation. Arginine supplementation has been used and recommended as a treatment for these acute attacks; however, insufficient evidence exists to support this treatment for MELAS. The mechanisms underlying the effect of arginine on MELAS pathophysiology remain unclear, although it is hypothesized that arginine could increase nitric oxide availability and, consequently, enhance blood supply to the brain. A more comprehensive understanding of these mechanisms is necessary to improve treatment strategies, such as dose and regimen adjustments; identify which patients could benefit the most; and establish potential markers for follow-up. This review aims to analyze the existing evidence concerning the mechanisms through which arginine supplementation impacts MELAS pathophysiology and provide the current scenario and perspectives for future investigations.

## 1. Introduction

Mitochondrial diseases constitute a heterogeneous group of genetic disorders characterized by defects in oxidative phosphorylation, caused by mutations in nuclear or mitochondrial DNA (mtDNA). These disorders exhibit a broad spectrum of clinical manifestations, affecting various organs, different age groups and varying degrees of severity. Among these conditions, one of the most prevalent is a syndrome characterized by mitochondrial encephalopathy, lactic acidosis and stroke-like episodes, known as MELAS [1]. The triad of lactic acidosis, seizures and stroke-like episodes is central for the diagnosis and pathogenesis [2]. However, patients with MELAS can also present other manifestations, such as migraine, cardiomyopathy, depression, cardiac conduction defects and diabetes, reflecting the multisystemic nature of this disease [1,2,3].

The genetic basis of MELAS has advanced with the identification of numerous mtDNA mutations associated with MELAS. However, the m.3243A>G (in the *MT-TL1* gene) is the most typical with this phenotype and found in approximately 80% of the cases [4,5]. Despite the advances in the molecular basis of MELAS, the mechanisms leading to the development of stroke-like episodes remain unknown [3,6].

Stroke-like episodes are recurrent cerebral insults that resemble strokes but are not considered to be of ischemic origin because the affected areas do not correspond to classical vascular distribution [1,2]. This irregular distribution of brain insults suggests a metabolic or small-vessel etiology [2]. There are three main hypotheses to explain the stroke-like episodes: (1) mitochondrial angiopathy, (2) mitochondrial cytopathy and (3) neuronal hyperexcitability [4,7,8,9]. The first hypothesis proposes that impaired mitochondria in small vessels would cause a microangiopathy, leading to inappropriate intracranial hemodynamics and stroke-like episodes. In the second hypothesis, a metabolic cytopathy would cause an imbalance between energy supply and demand, provoking neural damage and neuronal hyperexcitability [7]. The third hypothesis proposes that physiological changes in the cerebral cortex would trigger neuronal hyperexcitability and epileptic seizures, which would increase energy demand. The marked increased energy demand would cause an imbalance between the energy demand and supply, leading to neuronal dysfunction [8]. What triggers neuronal hyperexcitability is still unknown.

Considering the altered intracranial hemodynamics with a possible dysfunction in vasodilation, Koga et al. proposed a supplementation with L-ARG (L-ARG) as a treatment for the stroke-like episodes [10]. L-ARG is a semi-essential amino acid, but it can also act as a precursor of nitric oxide (NO^•^), a molecule with recognized vasodilator effect [10,11]. Increasing NO^•^ levels could enhance blood perfusion in the brain and other microvascular compartments, including muscle tissue. Furthermore, it was also proposed that the effect of L-ARG supplementation could not be restricted to stroke-like episodes but could also extend to symptoms such as migraine, muscle weakness, exercise intolerance and diabetes [12,13].

Clinical improvement has been observed with L-ARG given by intravenous infusions in the acute phase of stroke-like episodes and by oral supplementation as prophylactic treatment [10,14,15]. Good clinical outcomes were shown in several small studies and case reports. However, a recent systematic review concluded that there is not sufficient clinical evidence to support this therapeutic strategy in MELAS [16]. Nonetheless, L-ARG supplementation is frequently used in clinical practice, and it was recommended to MELAS patients by the Mitochondrial Medicine Society [17,18].

The mechanisms involved with the potential clinical improvement induced by L-ARG supplementation still need to be completely elucidated. Better clarification on these mechanisms is essential to improve treatment strategies, including dosage and regimen adjustments, identifying which patients would have the best beneficial effects, and establishing potential markers for follow-up.

This review aims to analyze the existing evidence concerning the mechanisms through which L-ARG impacts MELAS pathophysiology and provide the current scenario and perspectives for future investigations.

## 2. L-ARG

L-ARG is obtained from three different sources: diet, protein breakdown and endogenous synthesis via the intestinal–renal axis. Most of L-ARG originates from protein turnover (80%), while only 5–10% originates from the diet, and 10–15% is from the intestinal-renal axis [19]. The intestinal–renal axis is constituted by the epithelial cells of the small intestine and proximal tubule cells of the kidney. In the small intestine cells, citrulline is generated from glutamine. Citrulline is transported to the kidney, where L-ARG is produced through the action of the enzymes argininosuccinate synthase and argininosuccinate lyase [20].

**Figure 1:**
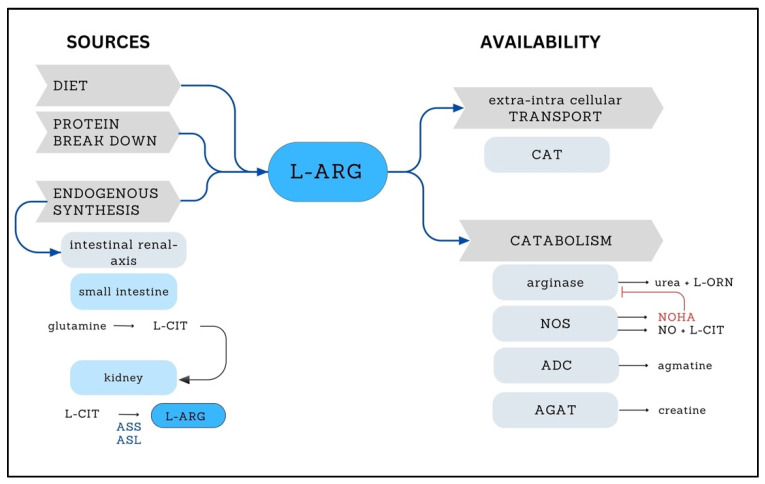
L-ARG sources and availability. The scheme shows the different sources of L-ARG and the processes that regulate its availability (gray arrow-shaped boxes). Bluish-gray rounded rectangles demonstrate the pathways or enzymes associated with these processes. Endogenous synthesis is performed by the intestinal renal axis with enzymatic steps occurring in the small intestine and kidney (light blue rounded rectangles). The enzymes involved in L-ARG transport and catabolism are shown in bluish-gray rounded rectangles. The red blunt arrow shows arginase inhibition by NOHA. L-CIT, L-citrulline; ASS, argininosuccinate synthase; ASL, argininosuccinate lyase; CAT, cationic amino acid transporter; L-ORN, L-ornithine; NOS, nitric oxide synthase; NOHA, Nω-hydroxy-L-ARG; NO, nitric oxide; ADC, arginine decarboxylase; AGAT, arginine/glycine amidino-transferase.

L-ARG availability also depends on its transport from the extracellular to the intracellular compartment and its catabolism through several enzymatic reactions (Figure 1). Like other amino acids, L-ARG requires specific transporters (cationic amino acid transporters) to cross the membrane into the cell. Once in the cell, L-ARG is used for protein synthesis and other reactions [21]. Deficiency of these transporters can also affect L-ARG availability and impact all L-ARG-dependent pathways.

In mammalian cells, L-ARG is catabolized by four enzymes: nitric oxide synthase (NOS), arginase, arginine decarboxylase and arginine/glycine amidino-transferase. These enzymes produce different metabolites: NO^•^ and L-citrulline by NOS; urea and L-ornithine by arginase; creatine by arginine/glycine amidino-transferase; and agmatine by arginine decarboxylase. L-ornithine is a precursor of glutamate and proline through the action of the enzyme ornithine decarboxylase and the L-Δ-pyrroline-5-carboxylate pathway, respectively [21,22,23]. In addition to producing NO^•^, NOS generates Nω-hydroxy-L-ARG, an arginase inhibitor [19] that modulates the availability of L-ARG to NOS [22] (Figure 1).

Hence, the regulation of L-ARG availability is complex and multifaceted, encompassing different cellular processes and enzymatic pathways. This complexity makes the investigation of L-ARG deficiency challenging.

## 3. Nitric Oxide Synthase

There are three well-characterized NOS isoforms: neuronal NOS, endothelial NOS and inducible. The neuronal and endothelial isoforms are constitutively expressed and regulated by the calcium–calmodulin system. Conversely, the inducible NOS is calcium-independent and activated by certain events, such as infection and inflammation [24,25,26]. NOS catalyzes the conversion of L-ARG to L-citrulline, producing NO^•^.

NO^•^ has different functions depending on the tissue and cell type. For example, NO^•^ is a vasodilator in blood vessels, it acts as a neurotransmitter in the nervous system and it plays a role in mediating immune responses [24,27]. In mitochondria, NO^•^ participates in the process of mitochondrial biogenesis and can regulate the respiratory chain by inhibiting Complex IV (cytochrome-*c*-oxidase). NO^•^ competes for the binding site of oxygen in cytochrome-*c*-oxidase, thus decreasing oxygen consumption and ATP synthesis [27,28,29,30]. This inhibition is reversible but can become irreversible with prolonged exposure to NO^•^, higher NO^•^ concentrations or when NO^•^ is converted to other reactive derivatives [28,31]. At the same time, as a reactive molecule, NO^•^ can also interact with other reactive oxygen species (ROS), promoting oxidative stress or participating in redox signaling. Therefore, the interactions of NO^•^ in the mitochondrial environment are highly complex, giving rise to diverse responses.

Additionally, NO^•^ synthesis can be regulated by L-ARG availability and inhibited by asymmetric dimethyl L-ARG (ADMA). ADMA is generated from protein hydrolysis after methylation of L-ARG residues and competes with L-ARG for the cationic amino acid transporters, limiting L-ARG entrance into the cell and reducing intracellular L-ARG availability [12].

## 4. L-ARG Levels in MELAS

When we consider a treatment based on the supplementation of L-ARG, the primary question to address is whether these patients have L-ARG deficiency. Koga et al. first reported this supplementation in MELAS, in a study on three patients with 16 stroke-like episodes, measuring the basal L-ARG level in blood from one patient [10]. In this patient, the L-ARG level was 100 ± 40 µmol/L on occasions separate from the stroke-like episodes. During acute attacks, the level dropped to 60 ± 10 µmol/L, suggesting that the decrease in the L-ARG level was related to the development of stroke-like episodes (Table 1). This relationship was supported by improved headache and clinical disability after incrementing L-ARG to 80 ± 10 µmol/L 24 h after supplementation. In 2005, this study was expanded to a larger group, with 24 patients and 72 controls, and showed that L-ARG levels were low in both acute and interictal phases and significantly lower in the acute phase [14]. Studies from other groups confirmed lower levels of L-ARG in adults [32,33] and children [34], except Naini et al., who did not find a significant difference between patients and controls [35]. The effectiveness of the dosage and regimen was confirmed by demonstrating the rise in L-ARG concentration after L-ARG supplementation during stroke-like attacks or interictal phase [10,11,32,33,34,36,37].

Not all the studies referred to the method used to measure serum L-ARG; thus, it is not possible to compare the values among the studies. However, it is interesting to note that, in most studies, the observed control values are compatible with the range from 80 µmol/L to 125 µmol/L, as mentioned in Evans et al. [38]. In the largest control group, with 72 subjects, the mean L-ARG level in the control group was 108 ± 28 µmol/L [30], similar to 94 ± 18 µmol/L (*n* = 4) in Rodan et al. (Table 1) [32,39]. A decreased L-ARG content was also found in cellular lysates from neuronal-like cybrid cell lines with the m.3243A>G [6], supporting the association of L-ARG deficiency with this mutation.

**Table 1:** L-ARG levels in the acute and interictal phases.

Ref.			Arginine (µmol/L)				
			Acute Phase		Interictal Phase		Controls
	Pat. (*n*)	Ctrl (*n*)	Basal	L-ARG	Basal	L-ARG	Basal
[10]	1	0	60 ± 10	80 ± 10	-	-	100 ± 40
[14]	24	72	47 ± 13 ^a,b^	92.4±15.9	84 ± 26	-	108 ± 28
[35]	10	10	81 ± 14				85 ± 19
[11]	15	20	36.4 ± 11.6 ^a,b,c^	101.7 ± 19.8	-	-	102.6 ± 15.7
[40]	24	72	46.6 ± 12.7 ^a,b,c^	-	83.6 ± 25.8	-	108.1 ± 27.6
[41]	1		46				27–108 *
[33]	10	10			57.1 ± 3.2 ^a,c^	143.8 ± 9.9	77.8 ± 4.4
[39]	3	4	-	-	53 ± 11 ^a^	76 to 230	94 ± 18
[34]	5	5	-	-	59 ± 5 ^a,c^	184 ± 14	151 ± 18
[42]	1	-	-	-	62.4 ± 2.7	-	-
[36]	10 ^$^; 13 ^#^	-	89.81 ± 76.25	143.44 ± 122.78	65.74 ± 16.74	162.36 ± 45.23	-
[37]	3	4	-	-	53 ± 11 ^a^	76 to 230	94 ± 18

*n*, number of patients; ^a^ significant (patient vs. control); ^b^ significant (acute vs. interictal); ^c^ significant (basal vs. after L-Arg); * reference values; ^$^ number of patients in the acute phase; ^#^ number of patients in the interictal phase; L-ARG, after L-ARG supplementation; Pat, patients; Ctrl, controls.

Some studies provided insights regarding the etiology of L-ARG deficiency, especially on L-ARG biosynthesis. Naini et al. suggested hypocitrullinemia as the cause of decreased NO^•^ production instead of L-ARG deficiency. Hypocitrullinemia could be caused by a deficiency in mitochondrial enzymes responsible for the biosynthesis of citrulline [35]. This idea was supported by other studies showing low citrulline levels in patients with MELAS [14,33,40]. One study did not find alterations in citrulline levels in the interictal phase, but the groups of patients and controls were smaller (three patients and four controls) [37].

Besides the low citrulline levels, El Hattab et al. observed an increased arginine clearance, which would be another explanation for an L-ARG deficiency [33]. Furthermore, in the same study, the de novo arginine synthesis rate was decreased in patients with MELAS [33]. These results are supported by a proteomic and metabolomic analysis that revealed the downregulation of the argininosuccinate synthase in patients with MELAS [43]. Argininosuccinate synthase is part of the L-ARG biosynthesis pathway. Thus, this result supports the hypothesis that the dysregulation of this pathway is the cause of L-ARG deficiency in MELAS. However, the study was performed with samples from a patient with a mutation in *MT-ND5*, so it should be confirmed in patients with the m.3243A>G mutation, which is the most prevalent in MELAS.

## 5. Effects of L-ARG Supplementation on NO^•^ Synthesis

Considering its role as a precursor for NO^•^ synthesis, the induction of NO^•^ production is frequently evaluated after L-ARG supplementation. The direct detection of NO^•^ in human samples is challenging. Therefore, researchers often measure NO^•^ metabolites, such as nitrate and nitrite (NOx), as an indirect measure of NO^•^. Nevertheless, few studies have measured NOx concentrations in patients and controls and have yielded conflicting results.

Considering only the basal levels, the study with the largest cohort (patients, *n* = 24; controls, *n* = 72) showed that the NOx level was decreased in the acute phase, as expected [20]. However, during the interictal period, NOx was increased [14]. In contrast, El-Hattab et al. did not find alterations in NOx levels between patients (*n* = 10) and controls (*n* = 10) during the interictal phase [33]. Similarly, Hanff et al. reported a patient with no alterations in NOx level considering a reference range [42]. Interestingly, with another approach, El Hattab et al. found that the NO^•^ synthesis rate was lower in adult and pediatric patients despite not identifying any abnormalities in NOx levels among adults [33,34]. These findings present contradictions, and a definite conclusion is challenging due to the limited number of studies and patients. Furthermore, only El Hattab controlled the diet, which could interfere with L-ARG concentrations [12].

Considering that NO^•^ production was decreased in patients, the levels of ADMA, a NOS inhibitor, were also measured [33,40]. El Hattab et al. found increased ADMA levels in patients with MELAS [33]. However, Koga et al. found no differences between controls and patients in the acute phase but found a higher ADMA/L-arginine ratio in patients in the acute and interictal phase [40]. Both studies suggest that NOS activity may be affected by ADMA in MELAS patients.

Investigations using different tissues or cells yielded inconsistent results regarding NO^•^ production. Studies with cybrids carrying the m.3243A>G mutation showed varied results concerning nitrite levels in distinct cell types. Glioblastoma-derived cells displayed no alterations [44], neuroblastoma-derived cells exhibited a decreased nitrite level [6] and osteosarcoma-derived cells demonstrated an increase in NO^•^ content [45,46]. Studies with skeletal muscle showed increased NOS activity in small muscle vessels and muscle fibers with mitochondrial proliferation in patients with the m.3243A>G mutation [45,47,48].

The studies assessing the impact of L-ARG supplementation on NO^•^ synthesis in patient samples showed variable results, but the methodologies applied differed. NO^•^ production was estimated indirectly by measuring NOx concentrations in plasma [14,33] or by determining the whole-body NO^•^ synthesis rate using stable isotope infusion [33]. Additionally, samples were collected at different time points, making comparisons difficult due to variations in L-ARG levels depending on the time elapsed after infusion or intake. Koga et al. observed that, during the stroke-like episodes, NOx concentrations increased after 15 min and 30 min of L-ARG administration but returned to interictal phase concentrations within 24 h. El-Hattab et al. found no significant increase in NOx measured 48 h after supplementation, while the NO^•^ synthesis rate increased after oral L-ARG during the inter-attack period [12,33]. In vitro studies evaluating the effect of L-ARG are scarce and are performed in different cell types and treatment conditions [6,46]. In one study, 24-h treatment with L-ARG decreased intracellular NO^•^ and protein nitration with no effects on Complex II or IV activities in homoplasmic mutant osteosarcoma-derived cells [46]. Another study revealed that a 3-day L-ARG treatment induced an increase in complex I activity and stabilized the formation of supercomplexes I + III in 100% mutant neuronal-like cybrid cells [6], suggesting that L-ARG could induce metabolic modifications directly affecting cellular mitochondrial function.

## 6. Effects of L-ARG Supplementation on Vascular Regulation and Hemodynamics

Vascular dysregulation due to impaired mitochondria is one possible explanation for stroke-like episodes in MELAS. Functional neuroimaging studies, including functional magnetic resonance imaging (MRI), magnetic resonance spectroscopy (MRS), single-photon emission computed tomography and positron emission tomography, offer valuable insights into cerebral hemodynamics in this disease. These methods allow for the evaluation of resting or stimulated cerebral blood flow and cerebrovascular reactivity, which measure the dilation response of blood vessels after vasodilatory stimulus [37].

Early studies demonstrated hyperperfusion (hyperemia) primarily in the infarcted areas during the acute phase of stroke-like episodes [49]. However, hyperperfusion was also observed in non-infarcted regions as a progression after the event [49], during the asymptomatic phase [50] or prior to the stroke-like episode [51]. The presence of hyperemia in an asymptomatic patient before the stroke-like event suggests that hemodynamic changes can occur months before clinical onset. Variable results can be explained by differences in clinical severity and the level of mtDNA mutant. These influences are supported by the correlation of hyperperfusion level with the severity of neurological phenotype and the percentage of mutant mtDNA [39].

It is hypothesized that hyperemia is a response to compensate for the metabolic imbalance caused by inefficient ATP generation if the flow control mechanism is preserved [52]. A second hypothesis is that hyperemia is the result of dysfunctional vasoregulation due to microangiopathy [53]. In this case, vasodilation would result from low pH due to lactate accumulation in smooth muscle cells of the vessels [8].

Abnormal responses to vasodilator substances were observed in stroke-like regions of MELAS patients but tended to normalize over time [49]. Interestingly, an inverse relationship between vasoreactivity (low vasoreactivity) and resting cerebral blood flow (high cerebral flow) was found in non-infarcted areas [49]. This type of response was explained by the vessels’ limited capacity to dilate further, either due to structural limitations or local lactic acidosis [49].

Functional brain imaging studies demonstrated that treatment with L-ARG altered hemodynamics in patients with MELAS. Rodan et al. studied two different brain regions: the occipital area, typically involved in stroke-like episodes; and the frontal area, as a non-involved region. L-ARG supplementation reduced cerebral blood flow in both regions, while cerebrovascular reactivity was affected differently, increasing in frontal areas and decreasing in occipital regions [37].

The use of functional MRI allowed for the evaluation of the impact of L-ARG supplementation on neural activation in response to visual stimuli. Patients had a reduced neural activation to a visual stimulus with no L-ARG; however, neural activation increased after a single dose of L-ARG or supplementation for six weeks [37]. These results suggest that L-ARG improved neural activation or neurovascular coupling response [37].

The involvement of neurovascular coupling in the pathogenesis of MELAS has recently been considered among the hypotheses explaining stroke-like episodes [37,54]. Neurovascular coupling is based on the concept of the neurovascular unit, comprising neurons, glial cells and cerebral vascular cells (vascular smooth muscle cells, pericytes and endothelial cells) [55]. Neurovascular coupling is the interaction of these three compartments (neuronal, glial and vascular), providing a mechanism for regulating cerebrovascular flow based on neuronal activity, and it is mediated by the glial compartment [56].

Our understanding of neurovascular coupling has expanded with its significance in cell signaling, cell–cell interactions and modulation by systemic factors such as blood pressure, glucose, metabolism and temperature [57,58]. It has been hypothesized that neurovascular uncoupling could be a contributing mechanism in various neurological diseases, including stroke, Alzheimer’s disease and autism spectrum disorders [59,60].

Using advanced MRI techniques can help us to understand the mechanisms involved in neurological diseases. Functional MRI can measure neuronal activity indirectly, detecting hemodynamic effects using blood oxygenation level-dependent responses [61]. Arterial spin labeling is another MRI technique that is able to measure blood flow, using magnetically labeled arterial blood water protons as an endogenous tracer [62]. Using a combination of the analyses of blood flow and regional brain activity, Wang et al. were able to evaluate neurovascular coupling [54]. They showed that neurovascular coupling was reduced in MELAS patients in the acute stage and that these abnormalities compromised other regions beyond the acute stroke-like lesions [54]. Additionally, they observed a trend of recovery after a 6-month follow-up. Given that L-ARG increases NO bioavailability, enhancing neurovascular coupling could be another mechanism of L-ARG’s effect on MELAS patients [63].

## 7. L-ARG Supplementation, the TCA Cycle and Mitochondrial Efficiency

Arakawa et al. proposed that L-ARG supplementation could also enhance the tricarboxylic acid (TCA) cycle by providing additional reducing equivalents to the oxidative phosphorylation, increasing ATP production [64]. L-ARG plays a multifaceted role in metabolic pathways [65]. Besides acting as a precursor to nitric oxide, L-ARG can be converted to L-ornithine, which, after several steps, generates α-ketoglutarate, an essential component of the TCA cycle [66] (Figure 1). Increased L-ARG availability accelerates the flow of the cycle [32,64] and increases the levels of intermediates [37,66]. On the other hand, L-ARG deficiency can compromise α-ketoglutarate replacement, worsening energy metabolism, particularly in MELAS cases [37].

Arakawa et al. were able to investigate TCA activity with positron emission tomography and a radiolabeled carbon acetate (C-11 acetate) in myocardial muscle [64]. With this method, the authors detected an increase in the TCA cycle activity in four of the six patients after L-ARG treatment, but with no increase in myocardial blood flow. These results suggested that L-Arg was not acting to promote NO^•^-mediated vasodilation [7,64]. In another study, Rodan et al. found increased levels of serum ornithine concentration after oral treatment with L-ARG in MELAS patients, which supported the hypothesis of enhancement of the TCA cycle by L-ARG [37].

Using MRS, a non-invasive method, it is possible to evaluate brain metabolism by detecting metabolites such as lactate and N-acetyl aspartate (NAA, marker of neuronal health) in patients with MELAS [67]. MRS usually demonstrates decreased NAA (amino acid synthesized in neuronal mitochondria), increased lactate (a marker of oxidative phosphorylation deficiency) and decreased glutamine/glutamate (neurotransmitters reflecting a decrease in neuronal spontaneous activities) in affected areas in acute stroke-like episodes [53,68].

Only two reports mention the effects of L-ARG on the MRS alterations [69,70]. Kubota et al. reported a patient who had five stroke-like episodes and received L-ARG infusion only during the fifth episode. Compared to the fourth stroke episode, MRS on the fifth episode showed a lower lactate peak and no alteration in the NAA level [69]. Hovsepian et al. studied another patient with serial MRS, 3 and 8 days after the infusion of L-ARG, during a stroke-like episode, and compared it to another exam, which was performed in a previous event without treatment with L-ARG that occurred 12 days before. They found that the lactate peak was lower and NAA higher in the episode treated with L-ARG [70]. However, the decrease in lactate and increase in NAA peaks, which suggest an improvement of mitochondrial function, cannot be conclusively attributed to L-ARG due to the lack of appropriate controls and the possibility that the alterations could be the natural evolution of the stroke-like event. Nevertheless, serum lactate was reduced after 24 h of treatment with L-ARG in 24 patients with MELAS compared to pre-treatment levels [14].

The effects of L-ARG on mitochondrial function were evaluated by in vitro cultured cells containing the m.3243A>G mutation [6,46]. Treatment of neuronal-like cells with L-ARG improved Complex I enzymatic activity in cells with 100% mutant DNA. However, the same improvement was not obtained in heteroplasmic cells with 70% mutant DNA. L-ARG increased Complex IV activity in control cells but not in cells containing the mutation, either homoplasmic or heteroplasmic [6]. An interesting finding of this study was that L-ARG stabilized the formation of supercomplexes I+III, which could improve cell metabolism and reduce ROS formation [6]. In another study, Barros et al. did not find improvement in Complex IV activity in osteosarcoma-derived cybrid cells and homoplasmic for the MELAS mutation [46]. However, these studies have different time treatments (24 h and 36 h) and cell sources, so further studies are necessary to clarify the specific effect of L-ARG on mitochondrial function.

## 8. Oxidative Stress in MELAS

Oxidative stress is thought to be involved in the pathogenesis of stroke-like episodes in MELAS. Increased ROS generation and decreased antioxidant enzymes were found in different types of patients’ samples, such as brain, skeletal muscle, skin fibroblast cells and cybrid cells containing the m.3243A>G [71,72,73,74]. Studies on brain samples obtained from patients with MELAS who died during the acute and subacute phases of a stroke-like episode showed an increased number of neurons with 8-OHdG (a marker for oxidative stress in DNA) [72]. A predisposition to oxidative stress was also demonstrated in other studies with decreased expression of antioxidant enzymes, such as SOD2 (MnSOD), catalase and glutathione in skeletal muscle and cybrid cells carrying the m.3243A>G [44,73,74]. The presence of oxidative stress was demonstrated with the findings of lipid peroxidation [73]. Reactive nitrogen species or nitrative stress was also detected in the intramuscular vessels of patients with MELAS [75] and in osteosarcoma-derived cybrid cells with the m.3243A>G mutation [46].

Ikawa et al. were able to demonstrate regional oxidative stress in brain lesions of stroke-like episodes non-invasively, using positron emission tomography [76]. They proposed that the hyperperfused brain would provoke an overloading of oxygen and enhanced glucose metabolism, causing an excess of electrons that, when added to the impaired respiratory chain, would increase the reactive species’ generation and result in oxidative stress [51].

To the best of our knowledge, there are no reports on the effects of L-ARG supplementation on oxidative stress in patients with MELAS. A study in cultured cells with the m.3243A>G mutation showed that L-ARG decreased nitrative stress [46]. Similarly, some studies with patients with arterial hypertension and diabetes supplemented with L-ARG supplementation showed an increase in the antioxidant capacity in blood samples [77,78,79].

## 9. Conclusions

**Figure 2:**
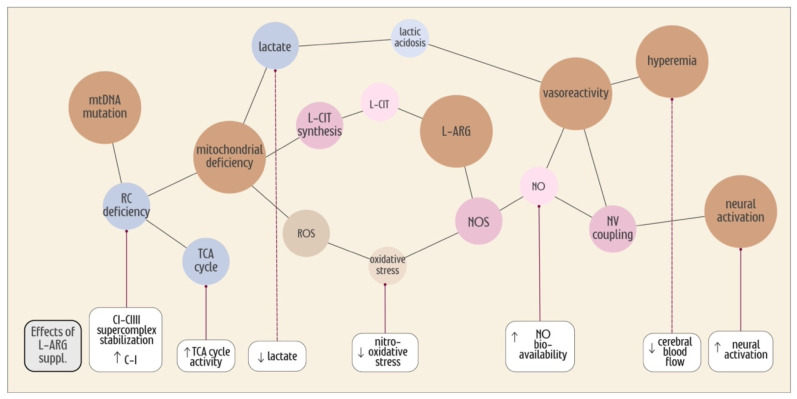
Key players in stroke-like development and possible effects of L-ARG supplementation. The figure demonstrates the complex connections (black lines) between the players (circles) involved in the development of stroke-like episodes in MELAS. The key players are shown in the brown circles. Other colored circles represent additional players related to oxidative stress (light brown), energy metabolism (blue) and NO pathways (pink). At the bottom, we show the effects of L-ARG supplementation (rounded-corner rectangles) found in different studies. mtDNA, mitochondrial DNA; RC, respiratory chain; suppl, supplementation; CI, Complex I; CIII, Complex III; TCA, tricarboxylic acid; L-CIT, L-citrulline; ROS, reactive oxygen species; NOS, nitric oxide synthase; NO, nitric oxide; NV, neurovascular.

More data in the literature are still needed to confirm the exact mechanisms through which L-ARG supplementation impacts MELAS patients. Arginine deficiency is well-documented during stroke-like episodes and interictal periods, but the underlying cause of this deficiency remains unknown. The metabolism and regulatory processes of L-ARG are highly complex and multifaceted, involving not only the NO^•^ pathways but also other processes relevant to neuronal activity, ATP production and oxidative stress (Figure 2). L-ARG supplementation effectively increases L-ARG serum concentration, impacting vasoreactivity with additional possible effects on neuronal activity and ATP production. Furthermore, these effects are not confined to the stroke-like regions but can also affect other brain regions.

The exploration of these mechanisms comes with various challenges. The rarity of this disease makes it difficult to conduct adequate clinical studies. Additionally, limitations on studying the brain in vivo, particularly during stroke-like episodes, and the lack of an animal model restrict more in-depth analyses of the potential pathways involved. Further investigations, especially on the effects of L-ARG on neuronal activity, neurovascular coupling and energetic metabolism, are still necessary, and clarifying these aspects can also benefit patients with other mitochondrial diseases.

## Data Availability

No new data were created or analyzed in this study. Data sharing is not applicable to this article.
